# Efficacy of blue-light blocking glasses on actigraphic sleep outcomes: a systematic review and meta-analysis of randomized controlled crossover trials

**DOI:** 10.3389/fneur.2025.1699303

**Published:** 2025-11-18

**Authors:** Francisco A. Luna-Rangel, Brenda Gonzalez-Bedolla, Miranda J. Salazar-Ortega, Ximena M. Torres-Mancilla, Salim Martinez-Cadena

**Affiliations:** 1 Tecnologico de Monterrey, School of Medicine and Health Sciences, Monterrey, Nuevo León, Mexico; 2 Tecnologico de Monterrey, School of Medicine and Health Sciences, Guadalajara, Jalisco, Mexico

**Keywords:** blue light exposure, sleep, actigraphy, circadian rhythm, crossover studies

## Abstract

**Background:**

Evening exposure to blue light suppresses melatonin, delays circadian phase, and prolongs sleep onset latency, impairing sleep quality. Blue-light blocking glasses (BBGs) are proposed as a non-pharmacological strategy to mitigate these effects, but trial evidence remains inconsistent due to small samples and heterogeneous protocols.

**Objective:**

To evaluate the efficacy of BBGs in improving objective sleep outcomes sleep onset latency (SOL), total sleep time (TST), sleep efficiency (SE), and wake after sleep onset (WASO) compared to clear lenses or no intervention in adults.

**Methods:**

A systematic search of PubMed, Scopus, and Web of Science identified randomized controlled trials (RCTs) from 2010 to 2024. Eligible studies enrolled adults using BBGs before bedtime and reported actigraphy-derived outcomes. Random-effects meta-analysis was performed using the generic inverse variance method. The review was registered in PROSPERO (CRD420251034611).

**Results:**

Three double-blind crossover RCTs (*n* = 49) were included. BBGs showed a non-significant reduction in SOL (MD = −4.86 min; 95% CI: −20.23 to 10.52; *p* = 0.54) and a non-significant increase in TST (MD = 8.75 min; 95% CI: −35.31 to 52.82; *p* = 0.70). No significant effects were found for SE (MD = −0.61; 95% CI: −7.58 to 6.35; *p* = 0.86) or WASO (MD = −1.47; 95% CI: −14.94 to 11.99; *p* = 0.83). Heterogeneity was low (*I*^2^ = 0%).

**Conclusion:**

BBGs may provide small improvements in sleep, but current evidence from RCTs does not support significant effects. Larger, well-powered trials with standardized protocols are needed.

**Systematic review registration:**

https://www.crd.york.ac.uk/PROSPERO/view/CRD420251034611.

## Introduction

In recent years, concern has grown about the impact of artificial lighting on sleep and circadian regulation, especially in the digital age. Widespread evening exposure to short-wavelength, blue-enriched light from electronic screens has been shown to suppress melatonin secretion, delay sleep onset, and alter circadian rhythms (1–4). These effects are particularly problematic in individuals with insomnia, circadian rhythm disorders, or irregular schedules, where nighttime light exposure further exacerbates sleep disturbances (5, 6).

Blue light, typically in the 460–480 nm range, is the primary stimulus for intrinsically photosensitive retinal ganglion cells (ipRGCs) containing melanopsin; when activated in the evening, they signal the suprachiasmatic nucleus (SCN), the master circadian clock, to inhibit pineal melatonin secretion (6, 7). As melatonin normally rises in the evening to facilitate sleep, its suppression by blue light delays circadian phase and increases sleep onset latency (8, 9). Experimental studies show that even short periods of evening tablet or smartphone use significantly reduce melatonin and shift its onset, resulting in later bedtimes and shorter sleep duration (1, 8). Thus, blue light after dusk exerts a potent alerting effect, reinforcing wakefulness and circadian misalignment; chronic exposure may cumulatively contribute to insufficient and irregular sleep, with adverse consequences for cognition, mood, and metabolic health (4, 7).

Accurate measurement of sleep is essential when evaluating interventions targeting circadian disruption. While polysomnography (PSG) remains the gold standard, its high cost, complexity, and artificial setting limit ecological validity. In contrast, actigraphy a wrist-worn accelerometer continuously monitors rest–activity cycles in real-world settings and provides reliable estimates of key sleep parameters such as sleep onset latency, total sleep time, and wake after sleep onset over extended periods (10, 11). Compared with sleep diaries, actigraphy avoids recall bias and improves objectivity; compared with PSG, it is unobtrusive, cost-effective, and feasible for long-term monitoring, although it may slightly overestimate sleep duration by misclassifying quiet wakefulness (12). Overall, actigraphy balances objectivity and practicality, making it especially suitable for assessing the ecological impact of interventions like blue-light blocking glasses.

Blue-light blocking glasses (BBGs) filter short wavelengths to reduce melanopsin activation in the evening, creating a “virtual darkness” that preserves endogenous melatonin secretion without requiring complete avoidance of artificial light (13, 14). They have gained popularity as a low-cost, behavioral strategy to improve sleep in individuals with insomnia, delayed sleep phase, or high evening screen exposure (15, 16). However, the evidence regarding their efficacy remains inconsistent. Some randomized controlled trials report improvements in subjective sleep quality and objective outcomes like earlier sleep onset or longer total sleep time (15, 17, 18). A 2020 meta-analysis found modest benefits on total sleep time and sleep efficiency, with larger improvements in self-reported sleep quality (5). More recently, a Cochrane review concluded that evidence remains inconclusive: approximately half the trials showed benefits, while the rest did not (19). Additionally, Liset et al. (13) reported no significant actigraphic sleep improvements with BBGs among healthy pregnant women, suggesting that sample characteristics and baseline sleep quality may moderate effects.

The efficacy of BBGs may also depend on the spectral filtering properties and usage protocols. Glickman et al. (20) introduced a novel metric melanopic daylight filtering density (mDFD) to quantify filter effectiveness, finding wide variability among commercially available glasses and emphasizing that only filters with mDFD ≥1 offer sufficient reductions in melanopic input to justify the “blue-blocking” label. Complementing these findings, several studies highlight the broader role of light timing and spectrum on sleep and circadian rhythms. Hand et al. (21) showed that more regular exposure to light across 24 h is associated with greater stability of sleep patterns in adolescents, while Ricketts (22) reviewed evidence that evening exposure to electric light suppresses melatonin, delays sleep onset, and reduces sleep quality, particularly in this vulnerable population. Similarly, Kim and Casement (23) emphasized that ensuring adequate access to daylight during the school day improves circadian alignment and supports sleep health, underscoring the importance of spectral composition as well as timing of exposure. Finally, Rynders et al. (24) provided naturalistic actigraphy data showing how changes in light exposure during the COVID-19 pandemic were linked to shifts in adolescent sleep and activity patterns. Taken together, these findings reinforce that the effects of BBGs cannot be considered in isolation but must be interpreted within the broader context of daily light exposure patterns and individual circadian vulnerability.

Despite increasing BBG use in clinical and consumer settings, no systematic review has exclusively focused on randomized crossover trials using actigraphy the methodologically rigorous design best suited to isolate individual-level effects under real-world conditions. This gap limits clarity around whether BBGs are a truly effective, low-risk strategy to mitigate modern light-induced circadian disruption. Therefore, the objective of this systematic review and meta-analysis is to evaluate the impact of blue-light blocking glasses on actigraphy-based sleep parameters in adults, compared to clear lenses. The primary outcomes of interest are sleep onset latency (SOL), total sleep time (TST), sleep efficiency, and wake after sleep onset (WASO), which reflect core aspects of sleep initiation, duration, and continuity. By synthesizing evidence from the past decade, we aim to determine whether BBGs yield meaningful improvements in these outcomes and to provide guidance for clinicians and researchers concerned with sleep health in the digital era.

## Materials and methods

### Study design

This systematic review and meta-analysis was conducted following the PRISMA 2020 guidelines (13) and was prospectively registered in the PROSPERO database (CRD420251034611) (16). A systematic review with quantitative synthesis was chosen as the most appropriate approach to address our research question, given the small sample sizes, methodological variability, and inconsistent findings across individual trials evaluating blue-light blocking glasses (BBGs). By pooling data, this design allows for increased statistical power, improved precision of effect estimates, and a more comprehensive and unbiased assessment of the available evidence.

The primary objective was to evaluate the efficacy of BBGs in improving sleep outcomes measured objectively through actigraphy. Eligible studies were required to report at least one actigraphic sleep outcome, such as sleep onset latency (SOL), total sleep time (TST), sleep efficiency, or wake after sleep onset (WASO).

### Participants

Studies recruited adult participants (≥18 years) from diverse backgrounds. Eligible populations included both healthy individuals with good baseline sleep quality and individuals experiencing symptoms of chronic insomnia or other sleep disturbances. By allowing both healthy and sleep-disturbed populations, the review aimed to capture a broad spectrum of evidence on the potential impact of blue-light blocking glasses on sleep outcomes. To ensure methodological consistency, all included studies were required to assess sleep using actigraphy.

Studies were excluded if they involved children or adolescents, individuals with severe psychiatric or neurological disorders unrelated to sleep, or participants using pharmacological sleep aids during the intervention period, as these factors could confound actigraphy-based sleep measures.

### Interventions

The intervention of interest was the use of blue-light blocking glasses (BBGs) designed to filter short-wavelength light exposure during evening or nighttime hours. Eligible studies included those evaluating amber- or orange-tinted optical lenses specifically intended to reduce blue-light exposure prior to sleep. Variations in duration of wear or specific timing before bedtime were accepted, as long as the primary mechanism involved optical filtration of blue light through glasses.

Studies were excluded if the intervention involved non-optical strategies to reduce light exposure, such as software-based screen filters, tinted contact lenses, or environmental light modifications (e.g., light bulbs or screen protectors).

### Comparators

Eligible comparators were clear-lens glasses that were visually indistinguishable from the active blue-light blocking glasses and worn during the same evening period, thereby preserving blinding and methodological consistency. This design was considered the most appropriate placebo condition to isolate the specific effects of short-wavelength light reduction. Studies were excluded if they did not include a clear-lens control, relied on screen-based software filters (e.g., f.lux or Night Shift), or lacked a counterbalanced condition.

### Systematic review protocol

The review protocol was developed *a priori* and prospectively registered in PROSPERO (CRD420251034611). Registration was undertaken to enhance transparency, reduce the risk of duplication, and provide a publicly accessible record of the intended methods. The protocol specified the research question using the PICOS framework, defined the eligibility criteria, and outlined the databases to be searched, the search strategy, and the types of outcomes to be extracted. It also prespecified the approach for data synthesis, including the use of meta-analytic models, planned subgroup analyses, and the methods to assess heterogeneity and publication bias.

All stages of the review were conducted in accordance with the Cochrane Handbook for Systematic Reviews of Interventions to ensure methodological rigor. Reporting was guided by the PRISMA 2020 statement to guarantee clarity and reproducibility.

### Search strategy

A comprehensive search strategy was designed combining controlled vocabulary terms (e.g., MeSH) and free-text keywords related to the intervention, outcomes, and study design. Boolean operators (AND/OR), truncation, and database-specific filters were applied to maximize sensitivity. The core search string included terms for blue-light blocking glasses (e.g., “Blue-light blocking glasses,” “Blue-light filtering glasses,” “Blue light filters,” “Amber lenses”), sleep-related outcomes (e.g., “Sleep quality,” “Insomnia,” “Circadian rhythm,” “Sleep latency,” “Total sleep time”), and study design (e.g., “Randomized controlled trial,” “RCT,” “Clinical trial”), while excluding studies in children or adolescents. The search was restricted to articles published in English and Spanish.

### Data sources

Searches were performed in PubMed/MEDLINE, Scopus, and Web of Science. The final search was completed on March 31, 2025. To ensure completeness, we also manually screened the reference lists of eligible articles and relevant reviews. Grey literature (e.g., conference abstracts, dissertations, unpublished data) was not included, in order to restrict the synthesis to peer-reviewed studies with sufficient methodological detail.

### Study sections and data extraction

Two independent reviewers (FALR and BGB) screened the titles and abstracts of all retrieved records, followed by full-text evaluation to determine final eligibility based on predefined criteria. Data extraction was performed using a standardized collection form designed to record sample size, participant demographics, study design, clinical or behavioral characteristics, intervention details (e.g., lens type, timing, duration), comparator conditions, actigraphic outcome measures (e.g., SOL, TST, SE, WASO), and numerical results for quantitative synthesis. Any disagreements were resolved through discussion and, if necessary, consultation with a third author (SMC), a board-certified neurologist with expertise in sleep research. Inter-rater agreement during the screening phase was evaluated using Cohen’s kappa coefficient to ensure consistency in study selection.

All records were managed using Rayyan (Qatar Computing Research Institute, Doha, Qatar), which facilitated blinded screening and resolution of conflicts. Extracted data were cross-checked for accuracy prior to analysis.

### Data analysis

Meta-analysis was planned using the generic inverse variance (GIV) method, appropriate for pre-calculated mean differences and standard errors. For crossover trials, when the standard deviation (SD) of the within-subject difference was not reported, the standard error (SE) was to be estimated assuming an intra-subject correlation of 0.5, as recommended in the Cochrane Handbook. While this estimation method is widely accepted, it introduces a degree of uncertainty because the true correlation may vary across studies and outcome measures.

A random-effects model was specified *a priori* to account for potential heterogeneity across studies. Effect sizes were to be expressed as mean differences (MD) with 95% confidence intervals (CIs). Statistical heterogeneity was to be assessed using the *I*^2^ statistic and the Chi^2^ test, with *I*^2^ values of 25, 50, and 75% considered low, moderate, and high heterogeneity, respectively. A *p*-value less than 0.05 was considered statistically significant.

When fewer than two comparable studies were available for a given outcome, findings were to be synthesized narratively. Publication bias was to be assessed using funnel plots and Egger’s test when ≥10 studies were available for an outcome. All analyses were planned to be performed using Review Manager (RevMan) version 5.4.1.

## Results

### Study selection and characteristics

**Figure 1:**
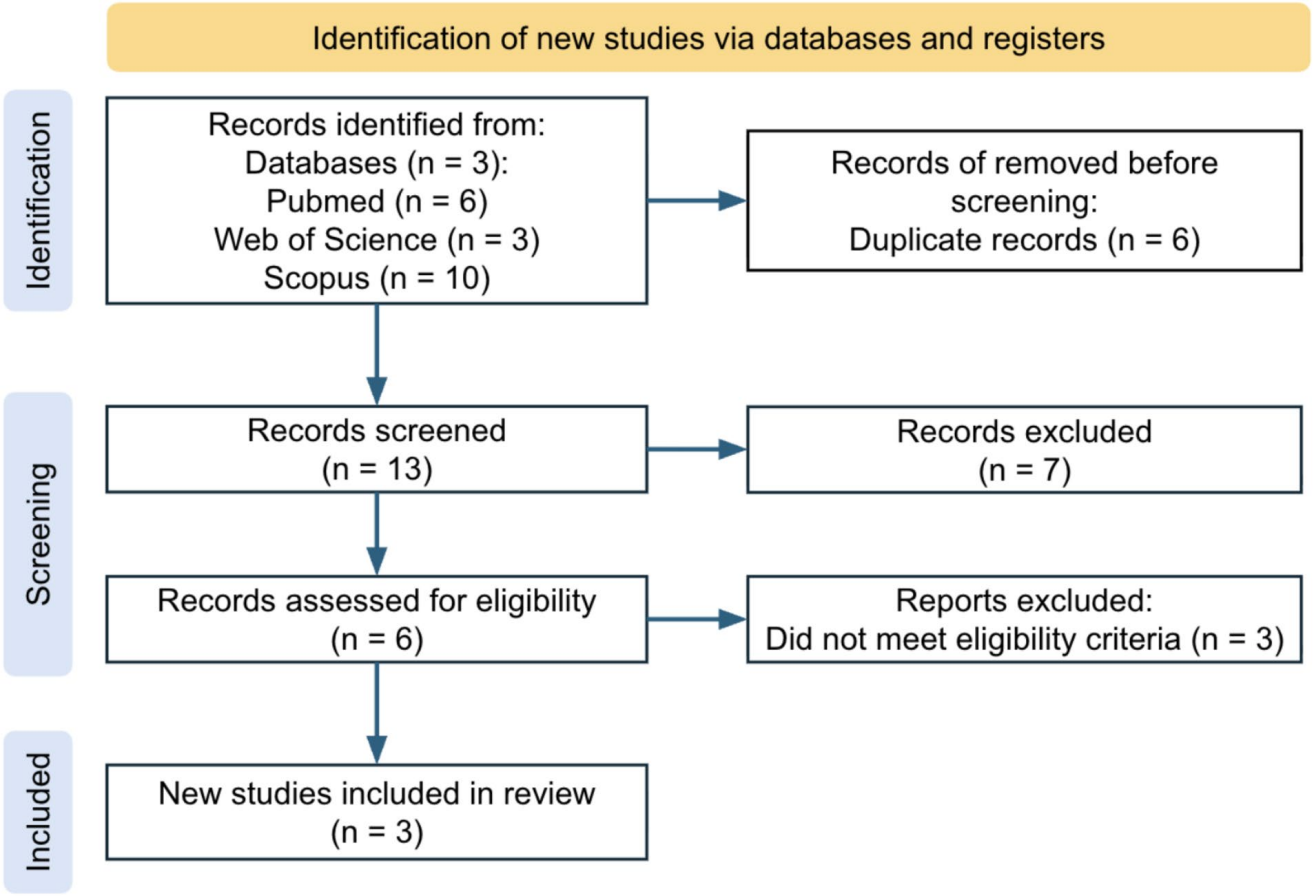
PRISMA flowchart illustrating the study selection process. A total of 19 records were identified through database searching (PubMed = 6, Web of Science = 3, Scopus = 10). After removing 6 duplicates, 13 records were screened, of which 7 were excluded. Six full-text articles were assessed for eligibility, and 3 met the inclusion criteria and were included in the review.

The initial database search yielded 19 records, of which 13 remained after duplicate removal. After screening titles and abstracts, 6 full-text articles were assessed for eligibility. Ultimately, 3 randomized controlled trials (7, 10, 18) met the inclusion criteria and were included in the final qualitative and quantitative synthesis. The remaining 3 full-text articles were excluded because they did not report actigraphy-based outcomes. A PRISMA flow diagram was constructed to summarize the screening and selection process (Figure 1).

Three randomized controlled crossover trials were included in this meta-analysis, each assessing the effects of blue-light blocking (BLB) glasses on objective sleep outcomes measured through actigraphy. All studies used a within-subject crossover design, allowing each participant to serve as their own control, and implemented washout periods between conditions to mitigate carry-over effects, although the duration varied across trials.

The total sample comprised 49 participants. Shechter et al. (14) studied 14 adults (57% female; mean age = 46.6 ± 11.5 years; BMI = 26.8 ± 4.2 kg/m^2^) who met clinical criteria for chronic insomnia symptoms, verified through the Insomnia Symptoms Questionnaire (ISQ). Knufinke et al. (25) recruited 15 recreational athletes (80% female; mean age = 23.3 ± 3.6 years) with no major sleep complaints. Bigalke et al. (15) included 20 healthy adults (45% female; mean age = 32 ± 12 years; BMI = 28 ± 4 kg/m^2^) with no diagnosed sleep disorders. Further demographic details are provided in Table 1.

**Table 1:** Population demographics.

Study	Population (*n*)	Age Mean ± SD	Male (*n*)	Female (*n*)	Body mass index
	Total	Total	Total	Total	Total
Shechter et al. (14)	14	46.0 ± 11.5	6 (43%)	8 (57%)	26.8 ± 4.2
Bigalke et al. (15)	20	32 ± 12	11	9	28 ± 4
Knufinke et al. (25)	15	23.27 ± 3.63	3	12	NA

SD, standard deviation.

Each study compared amber-tinted BLB lenses against visually indistinguishable clear-lens controls. Intervention protocols varied slightly: Shechter et al. (14) instructed participants to wear lenses for 2 h before bedtime over 7 consecutive nights; Knufinke et al. (25) applied a 3-h pre-sleep exposure over 9 nights; and Bigalke et al. (15) extended exposure from 6 p.m. until bedtime for 7 nights, following a 1-week baseline. Washout periods also differed: Shechter et al. (14) used a 4-week washout, Knufinke et al. (25) applied 4 days, and Bigalke et al. (15) incorporated a 1-week baseline period instead of a formal washout. Intervention protocols and design features are summarized in Table 2.

**Table 2:** Included articles characteristics.

Study	Study design	*n*	Participants	Intervention	Actigraphy device	Duration	Measured outcomes
Bigalke et al. (15)	Randomized controlled crossover trial	20	Healthy adults	1 week of blue light blocking glasses vs. 1 week of clear control glasses, worn from 6 p.m. until bedtime (after 1-week baseline)	Actiwatch Spectrum Pro, Philips Respironics, PA, USA	2 weeks	Self-report and actigraphy-based TST, SOL, WASO, number of awakenings and report (self-report) of sleep quality using the Karolinska Sleep Diary
Knufinke et al. (25)	Randomized controlled crossover trial	15	Adult recreational athletes with PSQI <7 and HSDQ <2.06	Amber-lens glasses (filtering short-wavelength light) vs. transparent glasses, worn 3 h before bedtime	Actiwatch 2, Philips Respironics, Murrysville, USA	9 consecutive nights	Actigraphy-based (lights-off and on, SOL, WASO, fragmentation index, TST, and sleep efficiency) Self-report (SOL, TST, WASO, KSS, number of awakenings)
Shechter et al. (14)	Randomized controlled crossover trial	14	Adults with chronic insomnia symptoms for >3 months, symptoms validated via the ISQ	Wearing amber-tinted blue light-blocking lenses vs. clear placebo lenses for 2 h before bedtime	ActiLife LLC, Pensacola, FL, USA	7 consecutive nights	PIRS score, daily post dairy (bedtime, wake time, time at which lenses were worn), PSQI (estimates of SOL, TST, WASO), actigraphy-based sleep estimates

PSQI, Pittsburgh Sleep Quality Index; HSDQ, Holland Sleep Disorder Questionnaire; ISQ, Insomnia Symptoms Questionnaire; KSS, Karolinska Sleepiness Scale.

Sleep outcomes were measured using validated wrist-worn actigraphy devices: the wGT3X-BT Actigraph (ActiLife LLC, Pensacola, FL) in Shechter et al. (14), the Actiwatch 2 (Philips Respironics) in Knufinke et al. (25), and the Actiwatch Spectrum Pro (Philips Respironics) in Bigalke et al. (15). These devices captured total sleep time (TST), sleep onset latency (SOL), sleep efficiency (SE), and wake after sleep onset (WASO), analyzed with standardized software (ActiLife or Actiware). All studies also included subjective assessments, such as sleep diaries or validated questionnaires (e.g., the Pittsburgh Insomnia Rating Scale), to complement objective actigraphy outcomes.

### Synthesized findings

**Figure 2:**
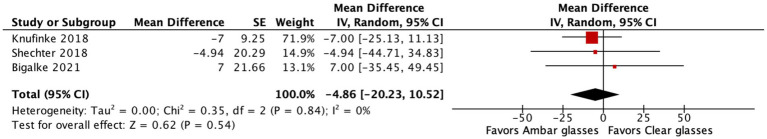
Forest plot showing the effect of blue-light blocking glasses (BBGs) versus clear lenses on sleep onset latency. The pooled mean difference (MD) was −4.86 min (95% CI: −20.23 to 10.52; *p* = 0.54), favoring BBGs but not statistically significant. Heterogeneity was low (*I*^2^ = 0%).

All three studies provided data for SOL. The pooled estimate showed a mean difference (MD) of −4.86 min [95% confidence interval (CI): −20.23 to 10.52], favoring BBGs over clear lenses (Figure 2). However, this difference was not statistically significant (*p* = 0.54). Heterogeneity across studies was low (*I*^2^ = 0%), indicating consistency in direction and magnitude of effects.

**Figure 3:**
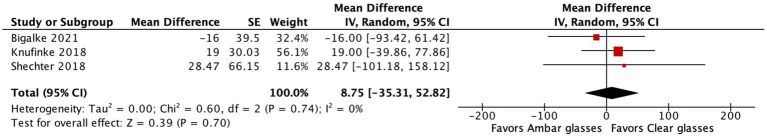
Forest plot of BBGs versus clear lenses on total sleep time. The pooled MD was +8.75 min (95% CI: −35.31 to 52.82; *p* = 0.70), indicating a non-significant increase with BBGs. Heterogeneity was low (*I*^2^ = 0%).

Data from all three studies revealed a non-significant increase in TST in the BBG condition, with a pooled MD of 8.75 min (95% CI: −35.31 to 52.82; *p* = 0.70) (Figure 3). Despite numerical favoring of the intervention, the wide confidence interval reflects substantial variability and low precision, likely due to small sample sizes.

**Figure 4:**
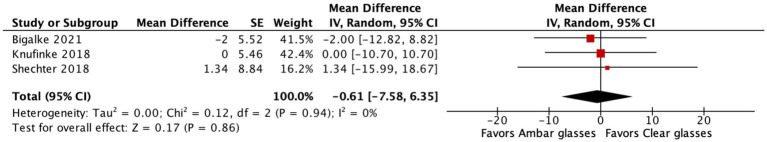
Forest plot of BBGs versus clear lenses on sleep efficiency. The pooled MD was −0.61% (95% CI: −7.58 to 6.35; *p* = 0.86), showing no significant difference between groups. Heterogeneity was negligible (*I*^2^ = 0%).

The pooled estimate for sleep efficiency showed a mean difference of −0.61 percentage points (95% CI: −7.58 to 6.35; *p* = 0.86), indicating no significant improvement associated with BBG use (Figure 4). Again, heterogeneity was negligible (*I*^2^ = 0%).

**Figure 5:**
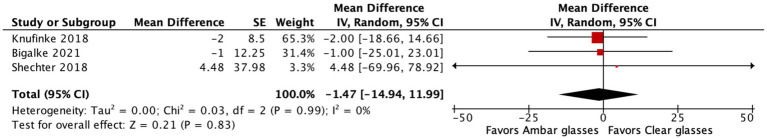
Forest plot of BBGs versus clear lenses on WASO. The pooled MD was −1.47 min (95% CI: −14.94 to 11.99; *p* = 0.83), indicating a small, non-significant reduction with BBGs. Heterogeneity was low (*I*^2^ = 0%).

For WASO, the pooled mean difference was −1.47 min (95% CI: −14.94 to 11.99; *p* = 0.83), suggesting a small, non-significant reduction in nighttime awakenings with BBGs (Figure 5). This outcome also showed no significant heterogeneity (*I*^2^ = 0%).

Across all outcomes, the effect directions generally favored BBGs, but none reached statistical significance. The consistency in results (*I*^2^ = 0% across analyses) suggests the intervention had uniform effects across populations and settings, albeit modest.

The extracted means and standard deviations for actigraphy-based sleep outcomes from the included studies are summarized in Table 3. These data were used to calculate effect sizes for each sleep parameter (TST, SOL, SE, WASO) in the meta-analysis. Differences refer to the change from the amber lens condition relative to the clear control condition.

**Table 3:** Data included in the meta-analysis.

Study	Actigraphic sleep onset latency			Actigraphic total sleep time			Actigraphic sleep efficiency			Actigraphic wakefulness after sleep onset		
	Ambar	Clear	Difference	Ambar	Clear	Difference	Ambar	Clear	Difference	Ambar	Clear	Difference
	Post	Post		Post	Post		Post	Post		Post	Post	
Shechter et al. (14)	11.27 ± 12.23	16.21 ± 23.42	−4.94 ± 20.29	358.80 ± 66.29	330.33 ± 66.01	28.47 ± 66.15	78.35 ± 9.00	77.01 ± 8.67	1.34 ± 8.84	88.43 ± 38.45	83.95 ± 35.53	4.48 ± 37.08
Bigalke et al. (15)	29 ± 25	22 ± 12	7 ± 21.66	433 ± 40	449 ± 39	−16 ± 39.50	83 ± 6	85 ± 5	−2 ± 5.52	40 ± 14	41 ± 11	−1 ± 12.25
Knufinke et al. (25)	12 ± 7	19 ± 11	−7 ± 9.25	469 ± 40	450 ± 36	19 ± 38.03	85.6 ± 5.1	85.6 ± 5.8	0 ± 5.46	11 ± 8	13 ± 9	−2 ± 8.50

Presents actigraphic outcomes from three studies, including sleep onset latency, total sleep time, sleep efficiency, and wakefulness after sleep onset, comparing amber versus clear lenses.

### Assessment of risk of bias

Risk of bias was assessed using the Cochrane Risk of Bias 2 (RoB 2) tool across five domains: (1) randomisation process, (2) bias arising from period and carryover effects, (3) deviations from the intended interventions, (4) missing outcome data, (5) measurement of the outcome, and (6) selection of the reported result.

Overall, the three included randomized crossover trials were judged to have either low risk of bias or some concerns, with no study rated as high risk in any domain. Specifically, all trials were judged to be at low risk for missing outcome data, measurement of outcomes, and selective reporting, supported by the use of validated actigraphy devices and standardized scoring protocols. Some concerns were raised regarding the randomisation process, as allocation methods were insufficiently described, and for deviations from intended interventions in certain studies, reflecting limited reporting on participant adherence.

Bias specific to crossover designs was also considered. Although all studies incorporated washout periods, the adequacy of these procedures varied, leading to a rating of “some concerns” in this domain. For example, Shechter et al. (14) implemented a four-week washout, Knufinke et al. (25) used a four-day washout, and Bigalke et al. (15) relied on a baseline period rather than a formal washout, without direct testing for residual carryover effects.

**Figure 6:**
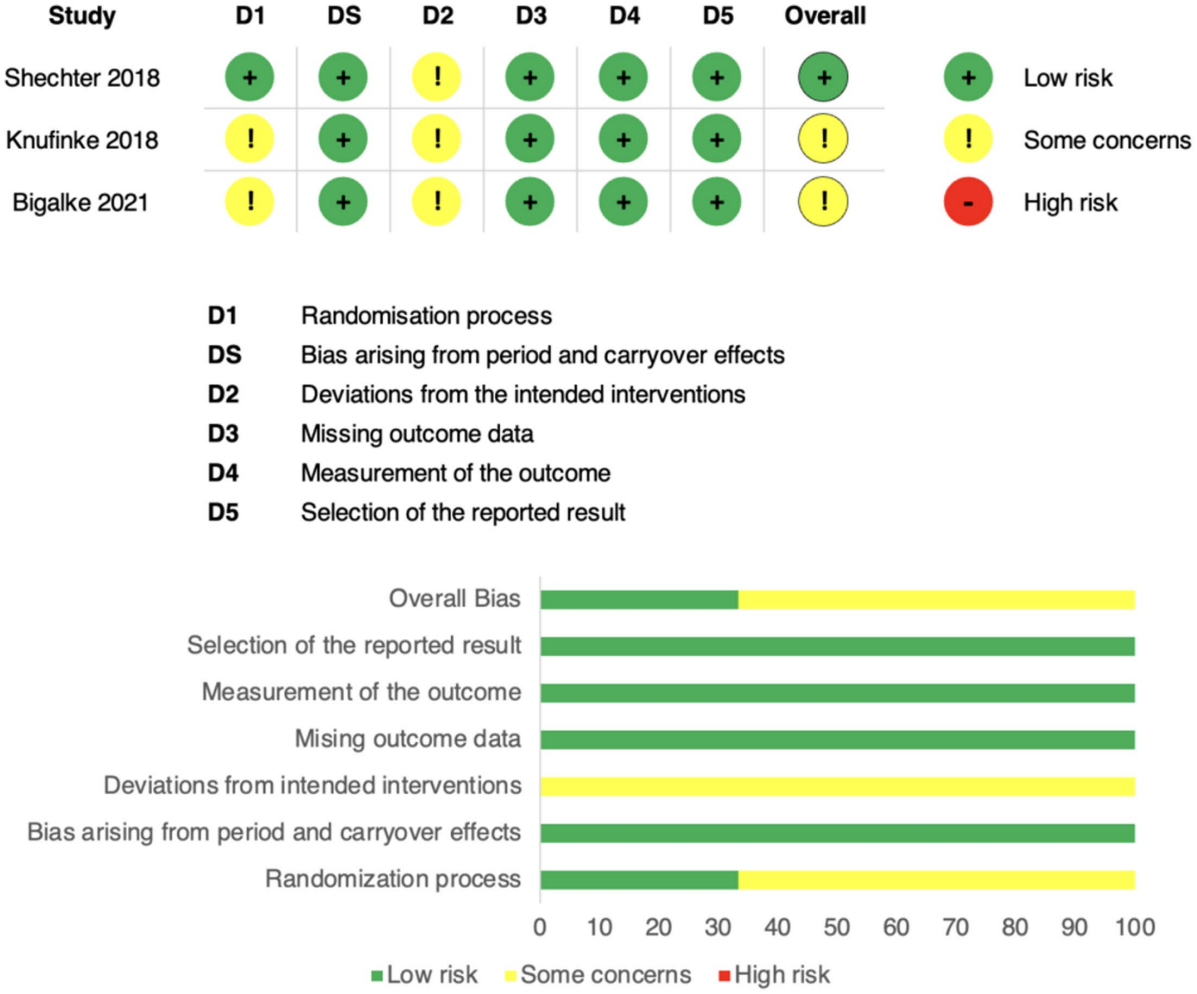
Risk of bias assessment using the Cochrane RoB 2 tool. Domains: D1 = randomisation process; DS = period/carryover effects; D2 = deviations from intended interventions; D3 = missing data; D4 = outcome measurement; D5 = selective reporting. Green = low risk, yellow = some concerns, red = high risk. The top panel shows study-level assessments; the bottom panel shows domain-level proportions.

Taken together, the body of evidence was considered to have an overall low risk of bias with some concerns, indicating adequate methodological rigor to support the reliability of the findings (Figure 6).

## Discussion

### Summary of main findings

The main finding of this meta-analysis is that BBGs, despite showing directionally favorable effects on actigraphy-based outcomes sleep onset latency (time required to fall asleep), total sleep time (overall duration of sleep), sleep efficiency (percentage of time in bed spent asleep), and wake after sleep onset (minutes awake after initially falling asleep) did not achieve statistically significant improvements. To our knowledge, this is the first meta-analysis focused specifically on actigraphy-derived sleep outcomes from randomized crossover trials, a design that reduces between-subject variability and provides a unique level of rigor compared to prior reviews.

The individual trials illustrate this pattern. In adults with insomnia, Shechter et al. (14) reported a modest increase in total sleep time, whereas Knufinke et al. (25) and Bigalke et al. (15) found no actigraphic benefits in recreational athletes and healthy adults, respectively, despite small subjective improvements. Parallel trials add perspective: Henriksen et al. (17) observed improved efficiency in mania, while Liset et al. (13) found no effect in pregnancy when using partially filtering controls. Esaki et al. (26, 27) reported signals of benefit in mood disorders, although actigraphy was not consistently the primary endpoint. Collectively, these findings suggest that BBGs may be most promising in clinical subgroups with circadian misalignment or evening hyperarousal, rather than in the general population.

Beyond sleep architecture, BBGs may influence other domains. For example, Zimmerman et al. (28) reported improved neurocognitive performance in insomnia patients using BBGs, despite minimal actigraphic changes. This raises the possibility that BBGs exert effects on alertness and cognitive function through circadian or arousal-related mechanisms not fully captured by actigraphy.

Our results also refine the conclusions of prior reviews. The Cochrane 2023 review judged evidence for BBGs as inconclusive and of low certainty. Shechter’s (5) meta-analysis suggested modest improvements, particularly in subjective outcomes, highlighting the gap between perceived and objectively measured sleep. Hester et al. (16) emphasized their potential in individuals with circadian misalignment, while Silvani et al. (6) confirmed that blue light reliably increases alertness but produces heterogeneous effects on sleep. Methodological commentaries, such as Glickman et al. (20), stress that only lenses with adequate filtering strength can meaningfully reduce melanopic light exposure at the eye.

### Limitations

This meta-analysis has several limitations that should be considered when interpreting its results. The crossover trials included were based on small samples and short intervention periods, generally about 1 week, which reduces statistical power and may underestimate longer-term effects. In addition, crossover designs present specific methodological challenges, particularly in sleep research, where natural night-to-night variability and potential carryover effects between treatment phases can confound outcomes. Inadequate washout periods and adaptation effects to the intervention may further obscure true treatment differences, thereby limiting the generalizability of the findings. Most participants were healthy adults with normal sleep patterns, which limits the ability to detect meaningful improvements and reduces the applicability of the findings to clinical populations. Although actigraphy is practical and ecologically valid, it may be less sensitive than polysomnography for detecting subtle changes in sleep architecture, and the studies employed devices from different manufacturers, which may have introduced variability in measurement accuracy. Moreover, none of the studies reported the actual melanopic light exposure at the eye, including intensity, spectrum, and timing, making it difficult to compare protocols or evaluate dose–response relationships.

Another important consideration is the potential for publication bias, as smaller studies with negative results may remain unpublished. In addition, the use of heterogeneous control conditions, such as partially filtering lenses, could have diluted true intervention effects. Participant characteristics also varied across studies: some trials included individuals with insomnia while others enrolled good sleepers, and the age range of participants differed substantially. Such variability may have influenced the magnitude of the observed effects, as participants without baseline sleep disturbances are less likely to experience significant improvement. Consequently, the results should be interpreted with caution, and future trials should stratify outcomes based on baseline sleep quality. Furthermore, both the actigraphy devices and the BBG lenses came from different manufacturers, introducing additional variability in measurement and intervention fidelity. These factors collectively introduce heterogeneity that may influence the observed effects and limit generalizability. Taken together, these limitations suggest that the current evidence should be interpreted with caution and highlight the need for larger, better standardized trials to clarify the role of BBGs in sleep regulation.

### Conclusions and future directions

In clinical practice, BBGs should be regarded as a low-cost and low-risk complement, not a substitute for evidence-based therapies. Broader expert consensus, such as that from the CIE, highlights the importance of bright, melanopic-rich light exposure during the day and reduced exposure in the evening. Within this framework, BBGs may help reduce evening light input. Other approaches, such as dimming room lighting, limiting evening screen use, or enabling warm “night modes” at reduced brightness, have also been proposed to minimize evening light exposure. Importantly, no clinical guidelines currently endorse BBGs as a standard intervention for insomnia or circadian rhythm disorders.

Future research should include larger and longer randomized trials, standardized reporting of spectral and melanopic light exposure, and the integration of BBGs into multicomponent interventions that combine environmental dimming, behavioral strategies, and digital tools. Ongoing clinical trials, such as the Phone Sleep Study (NCT05342662), bipolar mania (NCT01818622; NCT05206747), and studies combining BBGs with time-restricted eating (NCT06504342), will provide critical insights into their effectiveness.

A plausible hypothesis for future testing is that BBGs exert their greatest benefits when paired with behavioral interventions such as CBT-I or structured evening dimming, producing synergistic effects greater than either approach alone. Another avenue is exploring whether BBGs reduce cortical arousal via non-visual pathways, a mechanism that could be investigated using EEG or neuroimaging.

In conclusion, this meta-analysis shows that BBGs trend toward improving actigraphy-based sleep outcomes but without reaching statistical significance. When integrated with prior evidence, they appear most promising in specific clinical subgroups rather than in the general population. Until larger, standardized trials are available, BBGs should be considered a pragmatic adjunct, not a stand-alone therapy.

## Data Availability

The original contributions presented in the study are included in the article/Supplementary material, further inquiries can be directed to the corresponding author.
